# An Open‐Source, Reproducible MRI Data Acquisition and Reconstruction Workflow Using Pulseq: Multi‐Site and Cross‐Vendor Validation

**DOI:** 10.1002/mrm.70548

**Published:** 2026-08-06

**Authors:** Qingping Chen, Thomas H. M. Roos, Amaya Murguia, Yuhai Xie, Zheng Liu, Pengcheng Xu, Shohei Fujita, Sebastian Littin, Yogesh Rathi, Jiayu Zhu, Berkin Bilgic, Jon‐Fredrik Nielsen, Maxim Zaitsev

**Affiliations:** ^1^ Division of Medical Physics, Department of Radiology, University Medical Center Freiburg, Faculty of Medicine University of Freiburg Freiburg Germany; ^2^ Department of Radiology and Oncology, Center for Image Sciences University Medical Center Utrecht Utrecht the Netherlands; ^3^ Department of Electrical and Computer Engineering University of Michigan Ann Arbor Michigan USA; ^4^ Shanghai United Imaging Healthcare Co. Ltd. Shanghai China; ^5^ Wuhan United Imaging Life Science Instrument Co. Ltd. Wuhan China; ^6^ Athinoula A. Martinos Center for Biomedical Imaging Massachusetts General Hospital Boston Massachusetts USA; ^7^ Department of Radiology Harvard Medical School Boston Massachusetts USA; ^8^ State Key Laboratory of Extreme Optics and Instrumentation, College of Optical Science and Engineering Zhejiang University Hangzhou China; ^9^ Department of Radiology Juntendo University Tokyo Japan; ^10^ Department of Radiology The University of Tokyo Tokyo Japan; ^11^ Brigham and Women's Hospital Harvard Medical School Boston Massachusetts USA; ^12^ Harvard/MIT Health Sciences and Technology Cambridge Massachusetts USA; ^13^ Department of Biomedical Engineering University of Michigan Ann Arbor Michigan USA; ^14^ Functional MRI Laboratory, Department of Radiology University of Michigan Ann Arbor Michigan USA

**Keywords:** cross‐vendor MRI, data standardization, image reconstruction, open‐source technique, reproducibility, sequence development

## Abstract

**Purpose:**

Reproducibility in MRI is limited by variability in data acquisition, formatting, and reconstruction across sites and vendors. This work aimed to mitigate these challenges through an open‐source, vendor‐independent workflow originally developed for the 2023–24 ISMRM *Repeat It with Me: Reproducibility Team Challenge*.

**Methods:**

Pulseq, extended with advanced features including LABELs and Sequence Definitions for k‐space description and execution instructions, was used to harmonize data acquisition. Pulseq‐generated raw k‐space data were converted into the MRD format using these metadata. Open‐source, vendor‐independent image reconstruction and post‐processing were performed with Gadgetron. Additionally, vendor‐native online reconstructions on Siemens platforms (ICE and OpenRecon) were enabled for Pulseq‐based sequences. The workflow was validated using MPRAGE and EPI protocols in phantoms and in vivo across nine scanners from five sites and four vendors. Vendor‐native acquisition and reconstruction protocols were executed for comparison.

**Results:**

Pulseq‐based sequences executed successfully on all systems, with all data converted and reconstructed without errors. Pulseq‐based, Gadgetron‐reconstructed images showed good agreement with corresponding vendor‐native images across scanners; however, qualitative and quantitative differences remained. Despite detailed natural‐language descriptions and meticulous console configuration, vendor‐native protocols introduced subtle but notable cross‐platform differences in sequence execution and reconstruction. In contrast, the proposed workflow achieved highly consistent sequence execution via Pulseq and standardized reconstruction through Gadgetron across scanners.

**Conclusion:**

We established an open‐source MRI workflow that harmonizes data acquisition, formatting, and reconstruction across sites and vendors. This workflow represents a critical step toward fully open and reproducible MRI, facilitating transparency, standardization, and collaboration across the research community.

## 1 Introduction

Science advances incrementally through the development of theories and the formulation of testable predictions, gradually building and organizing knowledge. A cornerstone of this process is reproducibility—the ability to replicate or recreate scientific results—which is essential for validating findings and establishing robust evidence [1].

Magnetic resonance imaging (MRI) plays a crucial role in both scientific research and clinical practice, providing detailed images of internal body structures to detect and monitor diseases and assess treatment efficacy. While clinical MRI often relies on the expertise of radiologists visually reviewing images, there is an increasing imperative to improve reproducibility, transparency, and accessibility at all stages of MRI research, motivated by both the emergence of quantitative MRI and computer‐aided diagnosis. Strengthening reproducibility and lowering barriers to data and resource sharing would furthermore foster collaboration, enhance scientific rigor, and accelerate innovation.

However, achieving reproducibility in MRI remains challenging due to variability in data acquisition, data formatting and sharing, image reconstruction, and beyond. MRI data are typically acquired on proprietary commercial systems tied to specific vendors. Competitive, regulatory, and intellectual property constraints have led to closed or only partially open vendor‐specific hardware and software ecosystems over the decades. As a result, researchers must utilize such proprietary software environments to implement pulse sequences—a process that may demand years of specialized training. Moreover, expertise acquired on one vendor's system is seldom transferable to another vendor's system, which limits researcher mobility and hinders cross‐platform collaboration.

Consequently, variability in acquisition protocols across systems, sites, and even individual scanners undermines the reproducibility of scientific results, as differences in version‐ or vendor‐specific hardware and sequence configurations introduce inconsistencies. Although key sequence parameters such as repetition time and echo time are routinely reported, considerable variation persists in how sequences are implemented at a finer‐grained level, even when nominal high‐level parameters appear identical. Natural‐language descriptions and simplified sequence diagrams are insufficient to capture the technical detail required for faithful sequence reproduction. These limitations were highlighted in the 2020 T_1_ Mapping Reproducibility Challenge, which revealed that inter‐group variability in T_1_ measurements was twice that of intra‐group variability, even under strictly controlled conditions using standardized phantoms [2]. Harmonization across sites and vendors using vendor‐native sequences is achievable in principle, but it requires substantial effort and often leaves residual differences that cannot be eliminated. Jovicich et al. [3] demonstrated longitudinal reproducibility of resting‐state functional MRI using vendor‐provided sequences across multiple sites; yet they acknowledged persistent inter‐site differences in temporal signal‐to‐noise ratio driven by hardware and pulse sequence differences that could not be harmonized. More recently, Karakuzu et al. [4] showed that vendor‐neutral sequences significantly improved inter‐vendor reproducibility of quantitative MRI compared to vendor‐native sequences.

Furthermore, achieving true reproducibility requires public data sharing, which minimizes redundant imaging, enables the efficient reuse of existing datasets, and supports transparent communication to accelerate research dissemination and improve medical care [5]. However, raw data from vendor scanners are typically stored in proprietary formats that encode protocol parameters and reconstruction settings specific to each scanner software version. These formats, tightly coupled to the vendor's user interface, pulse sequence, and reconstruction architecture, are difficult to share and interpret outside their native environments [6]. The reluctance to adhere to standardized data formats further hampers cross‐platform data sharing and analysis. Even when k‐space data and trajectories are available as supplementary metadata, they often lack the complete context necessary for reproducible experiments [7]. Other barriers to data sharing include regulatory restrictions on human subject data, the cost of maintaining public repositories, and uncertainty regarding long‐term data accessibility.

At the image reconstruction stage, reproducibility is further hindered by factors such as variability in reconstruction algorithms, the lack of standardized pre‐processing and post‐processing pipelines, and insufficient reporting of computational methods. Vendor‐specific reconstruction software often functions as an opaque “black box,” giving researchers little insight into the processing steps applied to the data they receive. Data pre‐processing and post‐processing steps, such as image interpolation [8], low‐pass filtering [9], B1 bias field correction [10], and gradient nonlinearity correction [11], can all affect reproducibility; however, many of these processes remain proprietary and poorly documented. The impact of reconstruction variability was underscored in the 2019 ISMRM reproducibility challenge [12], which tasked teams with reproducing the results of the seminal SENSE‐based image reconstruction paper [13] using provided data. Despite visually minor differences in most submissions, substantial deviations in implementation details were observed. Seemingly minor details—such as maximum image intensity, field of view, or resolution—can lead to significant pixel‐wise discrepancies, even when images appear visually similar.

To alleviate these reproducibility challenges, we propose an open‐source, cross‐vendor, easy‐to‐learn workflow for efficient, transparent, and reproducible MRI data acquisition and reconstruction, built on the Pulseq [14] framework. Pulseq is an open‐source, vendor‐independent MR pulse sequence development platform that offers a user‐friendly, freely accessible foundation for open science and innovation. Prior studies have demonstrated its versatility in diverse applications, such as diffusion MRI [15, 16], X‐nuclei imaging [17, 18], radiofrequency (RF) pulse design [19, 20, 21], chemical exchange saturation transfer imaging [22, 23, 24, 25, 26], gradient coil design [27, 28, 29, 30], and low‐field MRI [31, 32].

In our workflow, Pulseq‐generated raw k‐space data from various software versions and vendors are standardized using the ISMRM Raw Data (ISMRMRD) [6] format, also known as the Magnetic Resonance Data (MRD) format, a community‐driven standard for storing and sharing raw k‐space data along with the physical acquisition parameters necessary for image reconstruction. These parameters are recorded via the LABEL and Sequence Definition [33] information embedded in the .*seq* files generated by the open‐source Pulseq software [34]. Building on the MRD backbone, we use Gadgetron [35] for open‐source, vendor‐independent, modular image reconstruction and post‐processing. Gadgetron also allows customized reconstruction via dedicated toolboxes and shared libraries. By integrating Pulseq and Gadgetron under the common MRD data standard, we establish a fully reproducible, end‐to‐end MRI workflow—from sequence design to final image reconstruction—promoting transparency, collaboration, and innovation in MRI research.

The present work was initially developed for the 2023–24 ISMRM *Repeat It with Me: Reproducibility Team Challenge* competition, where our *Pulseq Rocks* team won first place [36]. We validated it using two widely used sequences—Magnetization Prepared RApid Gradient Echo (MPRAGE) [37] and Echo‐Planar Imaging (EPI) [38]—across nine scanners from five sites and four vendors. Whereas vendor‐native protocols exhibited subtle but notable differences in sequence execution and reconstruction across platforms despite detailed documentation and configuration, our workflow achieved highly consistent sequence execution and standardized image reconstruction without errors across all systems. Moreover, we have demonstrated its applicability for establishing a quality assurance protocol for routine scanner performance monitoring, evaluating both image structural and temporal quality [39].

## 2 Methods

### 2.1 Workflow

The complete workflow is illustrated in Figure 1. It begins with sequence design using the advanced open‐source Pulseq software (v1.5.0), which supports high‐level programming in both MATLAB [34] and Python [40]. Sequences are exported in a vendor‐neutral .*seq* file format compatible with MR systems from Siemens [14], General Electric (GE) [41], Philips [42], United Imaging Healthcare (UIH) [43], and others (e.g., Bruker [44] and Canon [45]). This format is also supported by low‐field MR systems built on the open‐source MaRCoS electronic control system [31].

**FIGURE 1:**
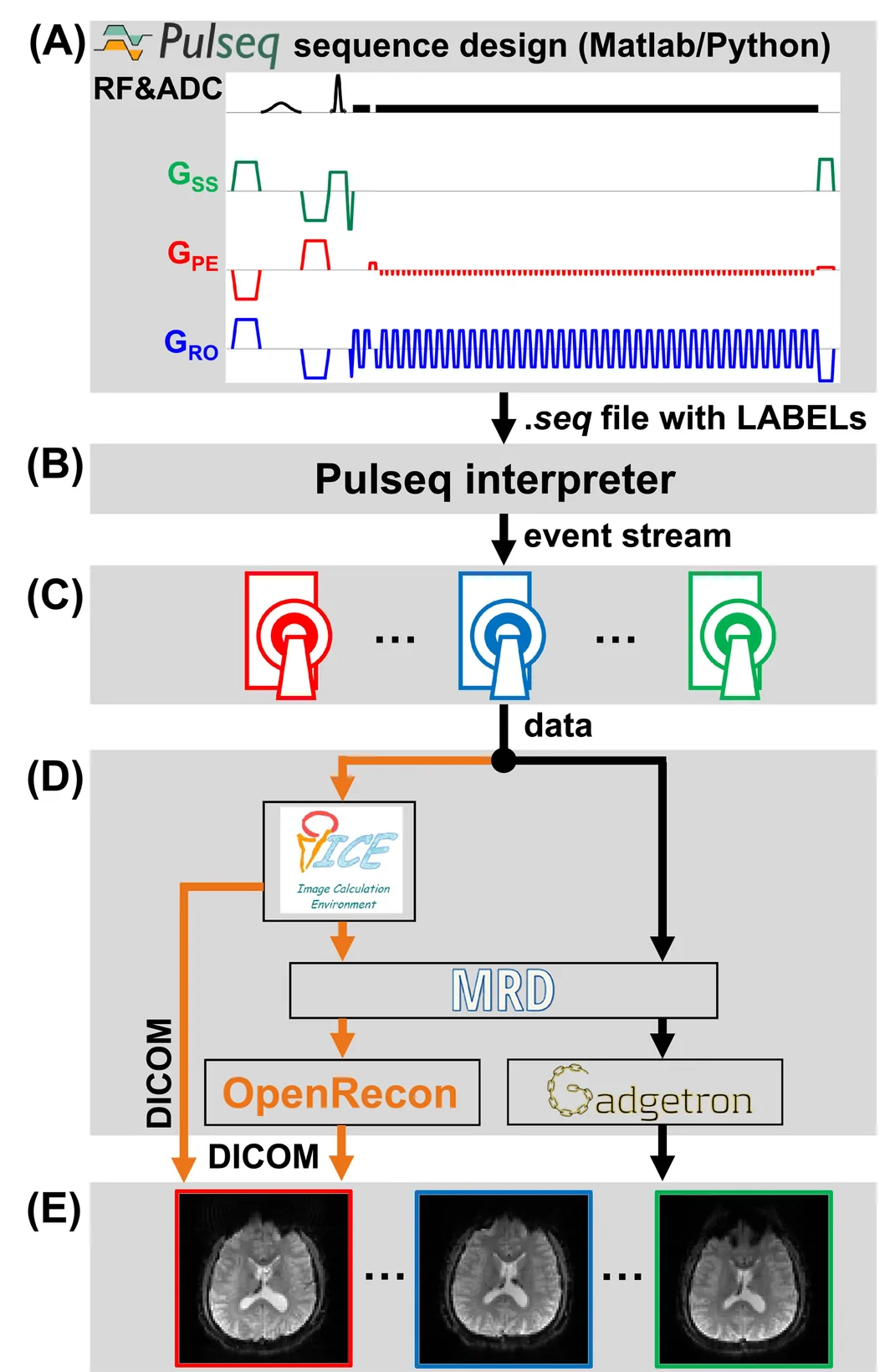
Overview of the complete workflow. (A) Example EPI sequence designed using the Pulseq MATLAB toolbox. (B) The vendor‐specific Pulseq interpreter loads the .*seq* file and streams sequence events in real time. (C) These events are executed on scanners from multiple sites and vendors. (D) Acquired raw k‐space data are either converted offline into the MRD format and reconstructed using Gadgetron, or streamed directly into ICE or OpenRecon for online reconstruction on Siemens platforms. (E) Final images are generated across sites and vendors using harmonized Gadgetron pipelines. Alternatively, ICE or OpenRecon sends DICOM images directly to the Siemens scanner console.

Next, a vendor‐specific Pulseq interpreter, typically implemented in a low‐level programming language such as C++, reads the .*seq* file and streams sequence events (e.g., gradient, RF, and analog‐to‐digital converter [ADC] events) for data acquisition on the scanner. The acquired raw k‐space data are subsequently converted offline into the vendor‐neutral MRD format. This conversion leverages LABELs [33] and Sequence Definitions embedded in the .*seq* file, which are used to label ADC events with appropriate k‐space counters and flags, and to instruct the interpreter on specific data‐acquisition behaviors.

Image reconstruction is then performed offline using Gadgetron, which processes MRD‐formatted data across vendors using harmonized, modular reconstruction pipelines. Alternatively, appropriately labeled raw data can be streamed line by line to Siemens' Image Calculation Environment (ICE) or OpenRecon (Siemens Healthineers AG, Erlangen, Germany) [46] frameworks. These two environments enable online reconstruction during data acquisition, automatically sending the resulting DICOM images directly to the scanner console. OpenRecon additionally supports the deployment of customizable, open‐source image reconstruction and post‐processing algorithms through an inline interface based on the MRD format, specifically for Siemens platforms [46].

### 2.2 Data Acquisition

MRI data were collected from both phantom and in vivo scans using nine MR scanners across five sites: Freiburg (Site 1), Boston (Site 2), Michigan (Site 3), Utrecht (Site 4), and Shanghai (Site 5). The dataset spans two magnetic field strengths (3 and 7 T) and includes systems from four major vendors: Siemens (Vendor A), GE (Vendor B), Philips (Vendor C), and UIH (Vendor D). Key acquisition details—including site, vendor, scanner model, software version, coil configuration, phantom/human subject specifications, and field strength—are summarized in Table 1. All human imaging was conducted with the approval of the local ethics committee. Written informed consent was obtained from all participants prior to their inclusion in the study.

**TABLE 1:** Detailed information on multi‐site, cross‐vendor data acquisition.

Site	Vendor	Scanner model	Software version	Coil	Phantom	Subject	*B*_0_ (T)
Freiburg, Germany (*Site 1*)	Siemens (*Vendor A*)	Trio TIM (*Scanner 1*)	VB19A	12‐channel head receive‐only coil (with the 4‐channel “CP mode”)	Structured liquid phantom doped with a NiCl/NaCl mixture	26 yo, male	2.9
		Prisma.Fit (*Scanner 2*)	VE11C, XA60A	20‐channel head receive‐only coil			
		Cima.X (*Scanner 3*)	XA61A	bioMatrix Head/Neck 20‐channel receive‐only coil			
Boston, USA (*Site 2*)	Siemens	Prisma.Fit (*Scanner 4*)	XA30A	32‐channel head receive‐only coil	Custom phantom (“readhead”)	34 yo, male	2.9
Michigan, USA (*Site 3*)	GE (*Vendor B*)	UHP (*Scanner 5*)	MR30.1_R01	32‐channel head receive‐only coil	ACR phantom	52 yo, male	3.0
Utrecht, the Netherlands (*Site 4*)	Philips (*Vendor C*)	Ingenia CX (*Scanner 6*)	Ingenia CX	32‐channel head coil	Spherical homogeneous liquid phantom	27 yo, male	3.0
		Achieva (*Scanner 7*)	Achieva	40‐channel head receive‐only coil	Spherical homogeneous liquid phantom	27 yo, male	7.0
Shanghai, China (*Site 5*)	UIH (*Vendor D*)	uMR 880 (*Scanner 8*)	V11_R3.2	32‐channel receive‐only coil	Structured liquid phantom	28 yo, male	3.0
		uMR Ultra (*Scanner 9*)	V12_R1	48‐channel head/neck receive‐only coil (without neck coil)	Structured liquid phantom	57 yo, male	3.0

Two widely adopted sequences, MPRAGE (Figure 2A) and EPI (Figure 2C), were implemented using Pulseq to ensure harmonized data acquisition across all scanners. Minor adjustments were made to accommodate system‐specific hardware constraints and spatial‐encoding conventions; unlike vendor‐native sequences, these modifications are fully transparent and documented [47]. Scanner‐specific hardware and timing constraints, as well as spatial encoding configurations for both sequences, are summarized in Table 2. Key sequence details are as follows:

- **MPRAGE**: includes a noise scan, water‐only RF excitation, and two‐fold acceleration via generalized autocalibrating partial parallel acquisitions (GRAPPA) [48] (Figure 2B). On Scanner 5 (Vendor B), the noise scan was acquired 5 s after the main sequence execution due to differing transmit/receive configurations. On Vendor C, an additional 0.5 ms delay was introduced between the noise scan and the subsequent inversion‐recovery RF pulse to comply with vendor‐specific timing constraints.
- **EPI**: incorporates a fat‐saturation module, ramp sampling for reduced echo spacing, and a three‐echo navigator for Nyquist ghost correction. On Scanner 1 (manufactured 18 years prior), the gradient spoiler amplitude was reduced due to hardware limitations. On the scanners from Vendor D, echo spacing was slightly increased from 0.70 to 0.72 ms due to a longer ADC dead time constraint.

**FIGURE 2:**
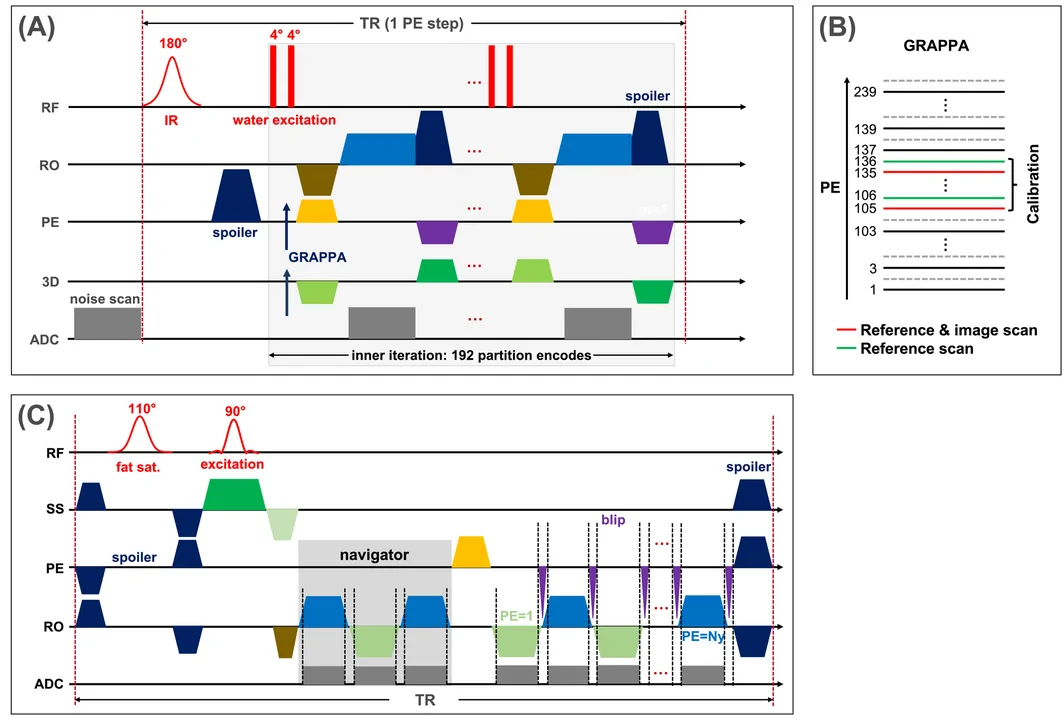
Sequence diagram and GRAPPA acceleration pattern. (A) Diagram of the Pulseq‐based MPRAGE. (B) Two‐fold GRAPPA acceleration pattern applied to the MPRAGE. Black solid lines represent acquired undersampled PE lines outside the calibration region, while gray dashed lines indicate skipped PE steps. The red and green lines correspond to reference lines used for GRAPPA calibration, with the red lines also contributing to the undersampled imaging data. (C) Diagram of the Pulseq‐based EPI. fat sat., fat saturation; IR, inversion recovery; PE, phase encoding.

**TABLE 2:** Summary of scanner‐specific hardware and timing constraints, along with spatial‐encoding configurations for the Pulseq‐based MPRAGE (sagittal orientation, phase encoding: From anterior to posterior) and EPI (transversal orientation, phase encoding: From anterior to posterior).

Scanner‐specific constraints	Vendor A	Vendor B	Vendor C	Vendor D
ADC/RF/gradient/block duration raster time (us)	0.1/1/10/10	2/4/4/4	0.1/1/10/10	0.1/1/10/10
RF/ADC dead time (μs)	100/10	100/0	100/10	100/35
RF ringdown time (μs)	10	60	10	10
MRPAGE's gradients	PE: +*k*_max_ ➔ −*k*_max_ RO: +*k*_max_ ➔ −*k*_max_ PA: −*k*_max_ ➔ +*k*_max_	PE: −*k*_max_ ➔ +*k*_max_ RO: +*k*_max_ ➔ −*k*_max_ PA: −*k*_max_ ➔ +*k*_max_	PE: +*k*_max_ ➔ −*k*_max_ RO: +*k*_max_ ➔ −*k*_max_ PA: −*k*_max_ ➔ +*k*_max_	PE: −*k*_max_ ➔ +*k*_max_ RO: +*k*_max_ ➔ −*k*_max_ PA: +*k*_max_ ➔ −*k*_max_
EPI's gradients	PE: +*k*_max_ ➔ −*k*_max_ RO: +*k*_max_ ➔ −*k*_max_	PE: −*k*_max_ ➔ +*k*_max_ RO: −*k*_max_ ➔ +*k*_max_	PE: +*k*_max_ ➔ −*k*_max_ RO: −*k*_max_ ➔ +*k*_max_	PE: −*k*_max_ ➔ +*k*_max_ RO: +*k*_max_ ➔ −*k*_max_
Slice‐selective RF frequency offset for EPI (Hz)	*G*_*ss*_ · *z*	−*G*_*ss*_ · *z*	*G*_*ss*_ · *z*	*G*_*ss*_ · *z*
Automatic readout oversampling	No	No	No	Automatic two‐fold readout oversampling
Maximum B1 field strength (μT)	∼20	25	25	∼21
Timing of noise scan	Right before the main sequence execution	5 s after the main sequence execution	0.5 ms before the main sequence execution	Right before the main sequence execution
Maximum gradient strength (mT/m)/slew rate (T/m/s)	Scanner 1: 40/200 Scanners 2 and 4: 80/200 Scanner 3: 200/200	Scanner 5: 100/200	Scanner 6: 80/200 Scanner 7: 40/200	Scanner 8: 80/200 Scanner 9: 100/200

*Note:* These constraints and configurations may vary across different scanners from the same vendor. All constraints, except the maximum B1 field strength and the maximum gradient strength, require slight code modifications.

Abbreviations: +*k*_max_ (−*k*_max_), the highest positive (negative) spatial frequency in k‐space along the corresponding spatial‐encoding direction; *G*_*ss*_, the amplitude of the slice‐selective gradient in (Hz/m); PA, partition encoding; PE, phase encoding; RO, readout; *z*, slice position in (m).

Corresponding vendor‐native sequences were carefully configured to match the Pulseq‐based implementations as closely as possible for comparison. Comprehensive sequence parameters for both implementations are detailed in Table 3. Note that the echo spacing of the vendor‐provided EPI sequences differs slightly across platforms, which may result in varying levels of distortion. The vendor‐provided EPI sequence from Scanner 7 (Vendor C, 7 T) exhibits a notably larger echo spacing (1.642 ms) compared to all 3 T platforms and is therefore expected to produce the most severe distortion.

**TABLE 3:** Parameters of the vendor‐native and Pulseq‐based MPRAGE and EPI protocols.

Seq.	Platform	FOV (mm^3^)	Acq. matrix size (voxel size (mm^3^))	Recon. matrix size (voxel size (mm^3^))	TR/TE/TI/TA (ms/ms/ms/ min:s)	RO BW (Hz/px)	Acc. mode	Fat sup.	Echo spacing (ms)
MPRAGE	Vendor A	256 × 240 × 192	256 × 240 × 192 (1 × 1 × 1)	256 × 240 × 192 (1 × 1 × 1)	2500/3.37/1100/5:38	200	GRAPPA	WE	9.18
	Vendor B	256 × 240 × 192	256 × 240 × 192 (1 × 1 × 1)	256 × 256 × 192 (1 × 1 × 1)	2604/2.18/1100/4:35	391	ARC	—	5.056
	Vendor C (3 T)	256 × 240 × 192	256 × 240 × 192 (1 × 1 × 1)	256 × 256 × 192 (1 × 1 × 1)	2500/4.21/1100/5:04	195	SENSE	WE	8.91
	Vendor C (7 T)^a^	256 × 240 × 192	256 × 240 × 192 (1 × 1 × 1)	256 × 256 × 192 (1 × 1 × 1)	2540/3.73/1100/5:09	195	SENSE	WE	8.23
	Vendor D	256 × 240 × 192	256 × 240 × 192 (1 × 1 × 1)	256 × 240 × 192 (1 × 1 × 1)	2500/3.7/1100/5:39	240	GRAPPA	WE	8.9
	Pulseq	256 × 240 × 192	256 × 240 × 192 (1 × 1 × 1)	256 × 240 × 192 (1 × 1 × 1)	2500/3.6/1100/5:40	195	GRAPPA	WE	8.94^b^
EPI	Vendor A	220 × 220 × 144	80 × 80 × 48 (2.75 × 2.75 × 3)	80 × 80 × 48 (2.75 × 2.75 × 3)	3630/33.0/−/1:53	1562	—	CHESS	0.7
	Vendor B	220 × 220 × 144	80 × 80 × 48 (2.75 × 2.75 × 3)	128 × 128 × 48 (1.72 × 1.72 × 3)	3656/32.3/−/2:08	6250	—	CHESS	0.468
	Vendor C (3 T)	220 × 220 × 144	80 × 79 × 48 (2.75 × 2.78 × 3)	80 × 80 × 48 (2.75 × 2.75 × 3)	3500/32.34/−/1:48	1869	—	SPIR	0.685
	Vendor C (7 T)^c^	220 × 220 × 144	80 × 79 × 48 (2.75 × 2.78 × 3)	80 × 80 × 48 (2.75 × 2.75 × 3)	3500/32.34/−/1:48	2240	—	SPIR	1.642
	Vendor D	220 × 220 × 144	80 × 80 × 48 (2.75 × 2.75 × 3)	80 × 80 × 48 (2.75 × 2.75 × 3)	3656/32.4/−/1:58	1570	—	CHESS	0.72
	Pulseq	220 × 220 × 144	80 × 80 × 48 (2.75 × 2.75 × 3)	80 × 80 × 48 (2.75 × 2.75 × 3)	3500/32.34/−/1:50	1563	—	CHESS	0.70 (0.72 on Vendor D)

Abbreviations: Acc. mode, acceleration mode; Acq. voxel size, acquired voxel size; ARC, autocalibrating reconstruction for Cartesian imaging (similar to GRAPPA); CHESS, chemical shift selective fat saturation; Fat sup., fat suppression method; FOV, field of view; GRAPPA, generalized autocalibrating partial parallel acquisition; Recon. matrix size, reconstructed matrix size; RO BW (Hz/px), readout bandwidth (Hz/pixel); SENSE, sensitivity encoding; Seq., sequence; SPIR, spectral pre‐saturation with inversion recovery; TA, total acquisition time; TE, echo time; TI, inversion time; TR, repetition time; WE, water excitation.

^a^ The MPRAGE sequence for the phantom measurement was set to be: “coronal” orientation; phase encoding direction = “left‐to‐right”; FOV = 256 × 192 × 240 mm^3^; acquisition matrix size = 256 × 192 × 240; acquisition voxel size = 1 × 1 × 1 mm^3^; reconstruction matrix size = 256 × 256 × 240; recon. voxel size = 1 × 1 × 1 mm^3^; TR/TE/TI/TA = 2540 ms/3.73 ms/1150 ms/5:09 min:s; readout bandwidth = 195 Hz/pixel; acc. mode = “SENSE”; Fat sup. = “WE”; echo spacing = 8.22 ms.

^b^ On Vendors A/B/C/D, echo spacing = 8.94/8.42/8.23/8.97 ms.

^c^ The reconstruction of the phantom echo‐planar images was interpolated: recon. matrix size = 192 × 192 × 48; recon. voxel size = 1.15 × 1.15 × 3 mm^3^.

### 2.3 MRD Conversion

Accurate, comprehensive scan metadata are essential for converting raw k‐space data to the MRD format, preserving the contextual information necessary for image reconstruction. To facilitate this conversion, we extended the Pulseq framework to embed LABELs and Sequence Definitions directly in the .*seq* files, providing the necessary scan metadata.

In our Pulseq‐based sequences, each ADC event was annotated with the LABEL metadata to encode key k‐space attributes. For example, an ADC event corresponding to the first phase‐encode line could be labeled using *mr.makeLabel(‘SET’, ‘LIN’, 0)*. Additional acquisition parameters, such as FOV, slice positions, slice thickness, inter‐slice gap, and readout oversampling factor, were specified through Sequence Definitions (e.g., *seq.setDefinition(‘FOV’, fov)*).

These LABELs and Sequence Definitions enable standardized MRD conversion and seamless integration with reconstruction frameworks such as ICE, OpenRecon, and Gadgetron for both online and offline reconstruction workflows [46, 49]. This approach promotes consistent interpretation of acquisition metadata across platforms, enhancing reproducibility, transparency, and interoperability in MRI research.

### 2.4 Image Reconstruction

Converted MRD data were reconstructed offline using the latest vendor‐independent, open‐source Gadgetron software, deployed in a Docker container. Gadgetron offers a modular suite of dedicated reconstruction toolboxes implemented as shared libraries. The following reconstruction pipelines were applied to both Pulseq‐based acquisitions (from all vendors) and Vendor A's product‐based data:

- **MPRAGE**: Reconstruction used the *Generic_Cartesian_Grappa.xml* configuration, which performed noise prewhitening, readout oversampling removal, coil compression, GRAPPA reconstruction, and image filtering.
- **EPI**: Reconstruction used the *epi.xml* configuration, which performed readout oversampling removal, EPI trajectory regridding, Nyquist ghost correction, coil compression, and magnitude reconstruction. Nyquist ghost correction was achieved via linear fitting of the phase difference between positive and negative readouts from the three‐echo navigator.

On Vendor A's systems, online reconstruction was enabled by extending Pulseq to integrate the vendor's reconstruction environments: ICE and OpenRecon [46, 49]. Online processing was achieved by configuring the measurement data header and other required scan parameters directly from LABELs and Sequence Definitions. In this study, all OpenRecon‐based images were contrast‐inverted relative to the corresponding ICE‐based images via an image‐to‐image application, a software tool for custom image post‐processing.

For comparison, vendor‐native online reconstructions for product sequences were configured to match Gadgetron as closely as possible across all vendors. To maximize reconstruction consistency, all additional console‐side data post‐processing steps (e.g., intensity correction, distortion correction, and image smoothing) were either fully or partially disabled. Reconstruction configurations for each vendor are summarized in Table 3.

### 2.5 Image Evaluation

Image comparisons were conducted to assess differences arising from various combinations of data acquisition and image reconstruction for both the MPRAGE and EPI protocols. These analyses included phantom and in vivo datasets across all vendors. To ensure consistency across reconstructions, ICE‐ and OpenRecon‐based images were shifted by one pixel along the phase‐encoding direction to align with Gadgetron reconstructions. Additionally, OpenRecon image contrast was re‐inverted to match ICE outputs.

For Vendor A, five comparison types were defined:

- Type A: Pulseq‐based versus vendor‐integrated acquisitions, both reconstructed with ICE. This comparison isolates sequence‐related differences while holding reconstruction constant.
- Type B: Pulseq‐based versus vendor‐integrated acquisitions, both reconstructed with Gadgetron. This comparison isolates sequence‐related differences using a vendor‐neutral reconstruction.
- Type C: Gadgetron versus ICE reconstructions for product‐sequence‐based acquisitions. This comparison isolates reconstruction‐related differences while holding acquisition constant.
- Type D: Gadgetron versus ICE reconstructions for Pulseq‐based acquisitions. This comparison isolates reconstruction‐related differences within the open‐source acquisition framework.
- Type E: Product‐sequence‐based, vendor‐reconstructed images versus Pulseq‐based, Gadgetron‐reconstructed images. This comparison captures the cumulative effect of adopting the full proposed workflow relative to the vendor‐native baseline.

For all other vendors, only Type E comparisons were performed.

Image difference was quantified using the following metrics within the phantom or head region [50]:

- Normalized root mean squared error (NRMSE): Measures the magnitude of difference between two images relative to the overall energy (L2 norm) of the reference image. Higher values indicate worse agreement.
- Structural similarity index (SSIM): Evaluates perceptual similarity based on local brightness, contrast, and structural information. Values close to 1 indicate higher similarity.
- High‐frequency error norm (HFEN): Quantifies differences in high‐frequency content (e.g., edges and fine structures). Higher values indicate reduced detail preservation.

In addition, normalized MPRAGE images were compared using intensity profiles (plots of intensity vs. position along a line through the image), and signal‐to‐noise ratio (SNR) was calculated for the echo‐planar images based on manually selected signal and background noise ROIs.

The Pulseq‐based sequence source code, MRD conversion scripts, step‐by‐step cross‐vendor data acquisition and reconstruction instructions, and example phantom data are publicly available online [51].

## 3 Results

The Pulseq‐based MPRAGE and EPI sequences were successfully executed across all scanner systems. All raw data acquired with Pulseq were converted to the MRD format and reconstructed using Gadgetron without errors. Representative images from Vendor A, illustrating different acquisition–reconstruction combinations, are shown in Figure 3. Corresponding quantitative evaluations across the five comparison types (A–E) are provided in Figure S1. Cross‐vendor comparisons between Pulseq–Gadgetron images and their vendor‐native counterparts are presented in Figures 4 and 5, with summary metrics shown in Figure 6. Complete metric values are listed in Tables S1 and S2.

**FIGURE 3:**
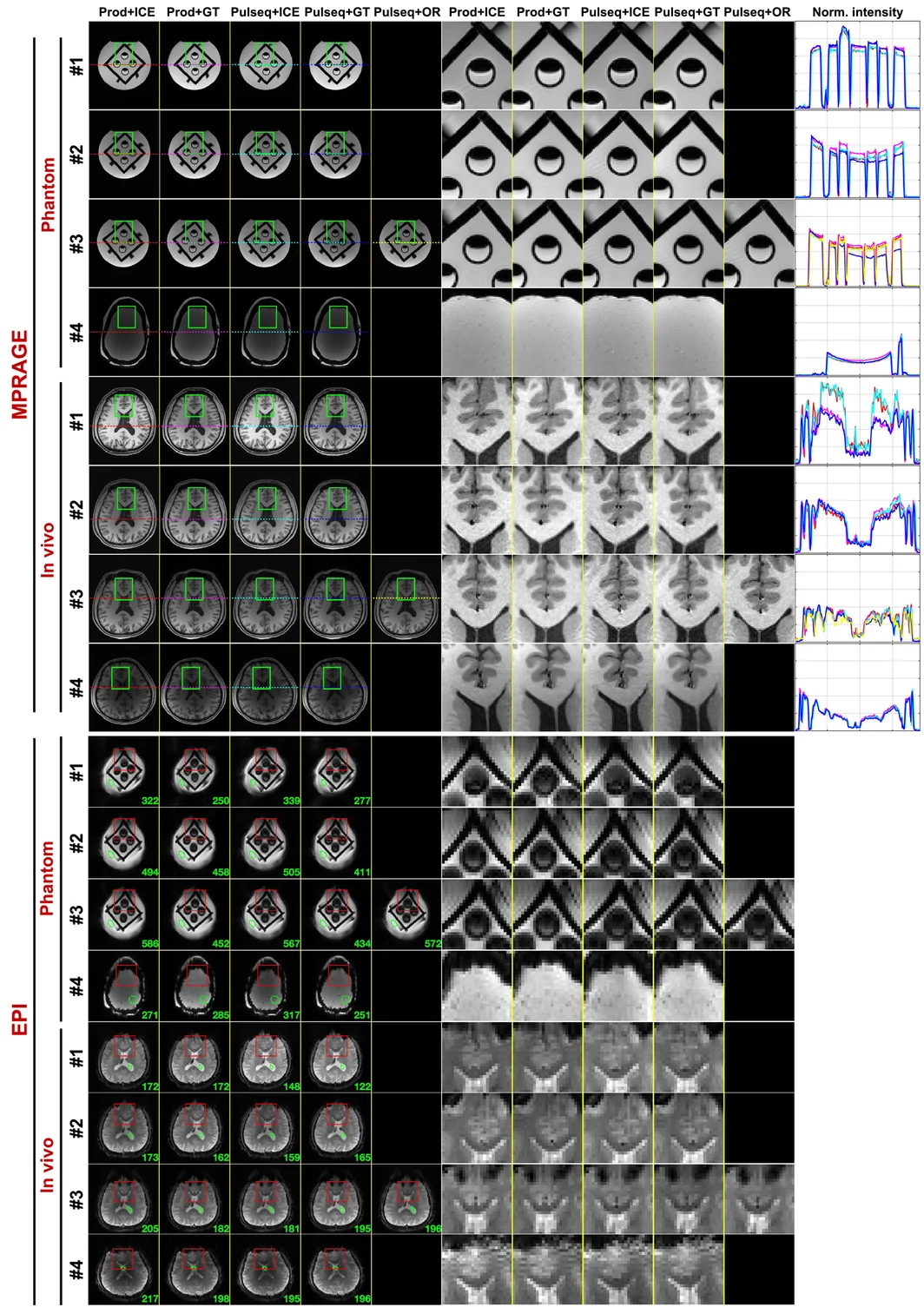
Representative MPRAGE and echo‐planar images acquired from phantoms and human subjects across all of Vendor A's platforms. Images were obtained using both product (Prod) and Pulseq‐based sequences and were reconstructed using three approaches: Vendor A's ICE reconstruction, Gadgetron (GT), and Vendor A's OpenRecon (OR). The “Norm. intensity” column shows intensity profiles from normalized MPRAGE images (plots of intensity vs. position along a line through the image). For echo‐planar images, the green number in the bottom‐right corner indicates the SNR value calculated from the manually selected ROI shown in green.

**FIGURE 4:**
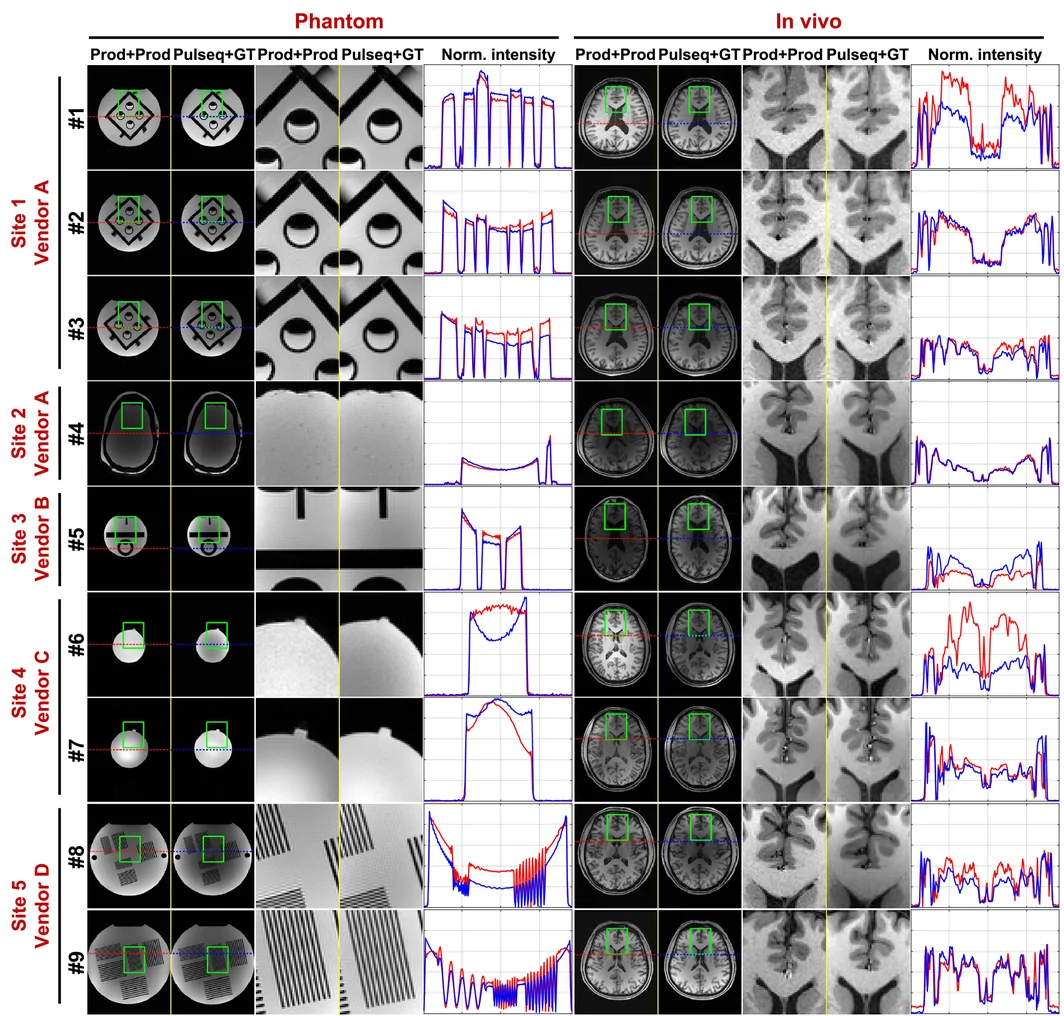
Representative MPRAGE images acquired from phantoms and human subjects across all participating sites and vendors. Images were obtained with Pulseq‐based sequences and reconstructed with Gadgetron. The “Norm. intensity” column shows intensity profiles from normalized images (plots of intensity vs. position along a line through the image).

**FIGURE 5:**
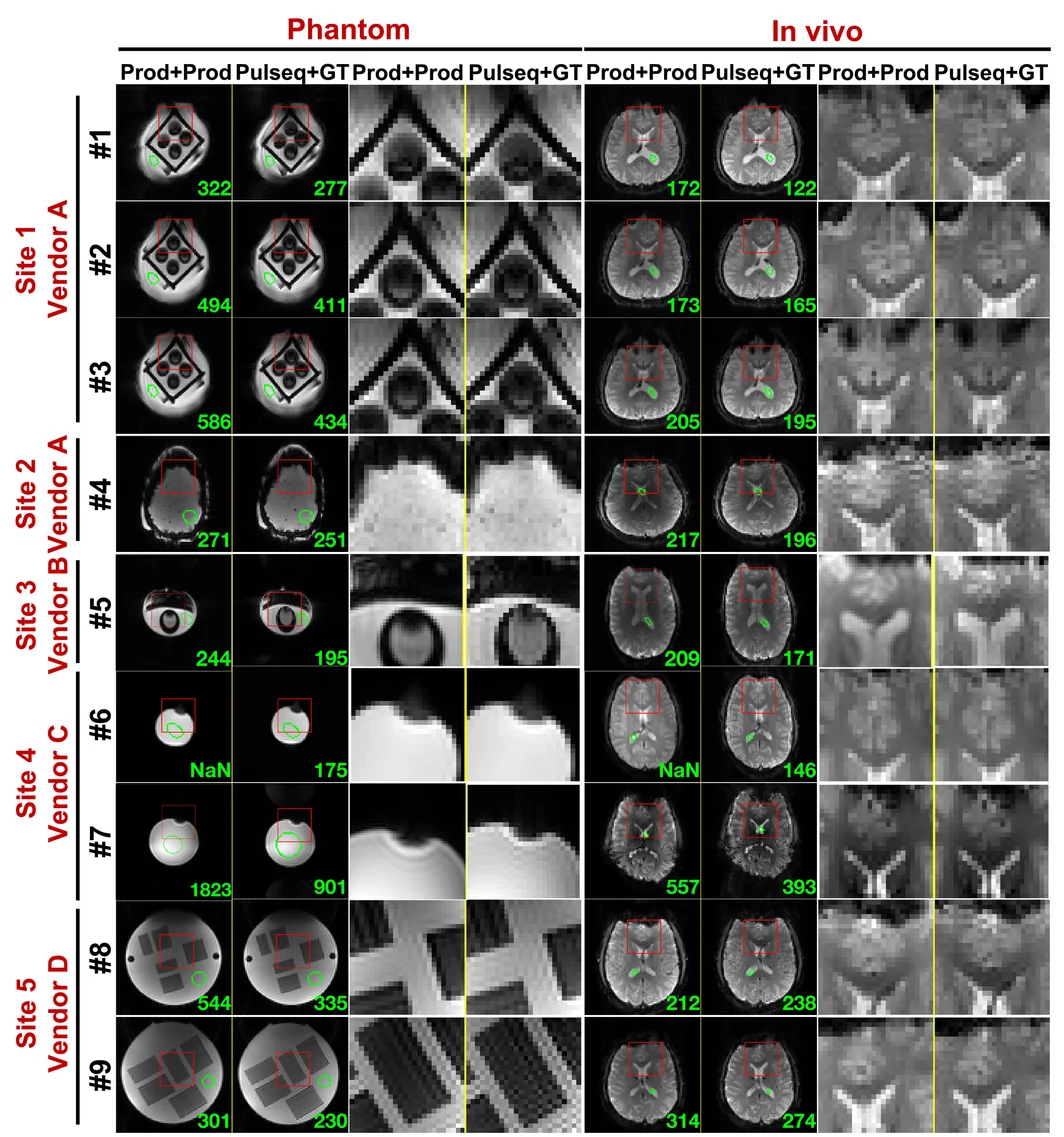
Representative echo‐planar images acquired from phantoms and human subjects across all participating sites and vendors. Images were obtained with Pulseq‐based sequences and reconstructed with Gadgetron. The green number in the bottom‐right corner indicates the SNR value calculated from the manually selected ROI shown in green. For Scanner 6, “NaN” is shown because the background noise was thresholded to zero in the DICOM images.

**FIGURE 6:**
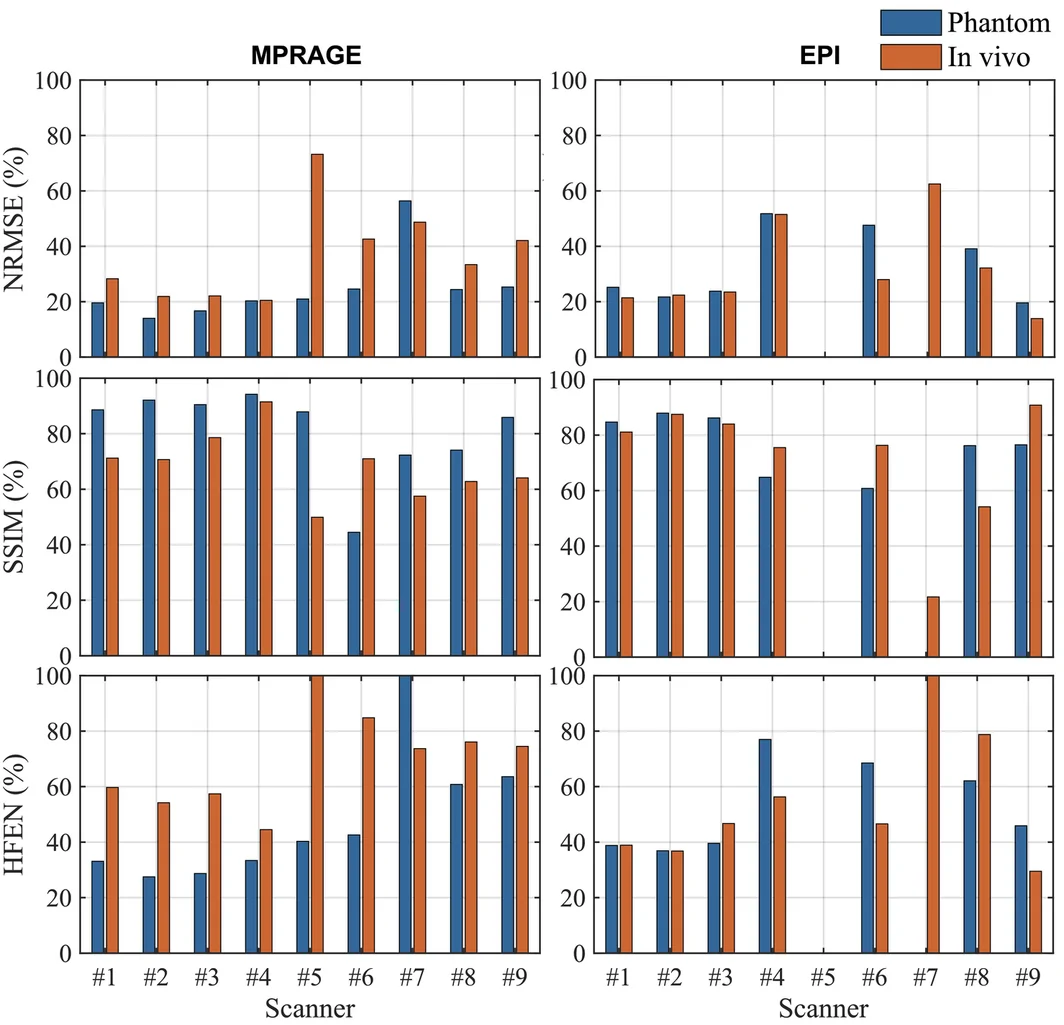
Quantitative analysis of image differences for MPRAGE and EPI in phantom and in vivo datasets across all scanners. Each panel displays evaluation metrics grouped by scanner model. Phantom and in vivo measurements are indicated in blue and red, respectively. SSIM values are calculated within the phantom or head region. Analysis was not performed for EPI scans on Scanner 5 and the phantom EPI scan on Scanner 7 due to differing matrix sizes. HFEN, high‐frequency error norm; NRMSE, normalized root mean squared error; SSIM, structural similarity index.

Visual comparisons across Vendor A systems show strong overall agreement among all acquisition–reconstruction combinations for both MPRAGE and EPI (Figure 3). For MPRAGE phantom scans, geometry and contrast are well preserved across methods, with only minor differences in edge sharpness and intensity uniformity. In vivo images exhibit greater variability, particularly within the highlighted ROIs, where differences in noise and local contrast are more pronounced. Re‐inverted OpenRecon images closely match the corresponding Pulseq–ICE images for both phantom and in vivo scans on Cima.X, confirming the effectiveness of OpenRecon for image post‐processing.

Normalized intensity profiles (MPRAGE) and EPI SNR values support these visual observations (Figure 3). For MPRAGE, normalized intensity profiles are generally comparable across acquisition–reconstruction combinations. In phantom scans, slight intensity differences are observed for Scanners 1–3. For in vivo images on Scanner 1, product‐sequence acquisitions show higher intensity in the white matter and cerebrospinal fluid than Pulseq‐based acquisitions. For EPI, Gadgetron reconstruction generally yields slightly lower SNR than ICE reconstruction, whereas Pulseq‐based acquisitions achieve SNR values comparable to those of product‐sequence acquisitions. Overall, Pulseq–Gadgetron echo‐planar images exhibit lower SNR than vendor‐native images for both phantom and in vivo scans.

Quantitatively, most comparisons yield low NRMSE (∼10%–30%), high SSIM (∼70%–95%), and moderate‐to‐high HFEN (∼20%–70%) (Figure S1). These trends indicate that global image structure is largely preserved, while differences are concentrated in high‐frequency components. For MPRAGE, phantom results generally outperform in vivo results, likely due to the phantom's simpler structure. In contrast, EPI shows phantom performance that is comparable or slightly worse, consistent with the more pronounced distortion artifacts in phantom images. Across scanners, comparison Types A and B consistently outperform Types C and D, suggesting that reconstruction differences contribute more strongly to variability than acquisition differences.

Across all vendors, Pulseq–Gadgetron images show good visual agreement with vendor‐native images for both MPRAGE (Figure 4) and EPI (Figure 5), supported by moderate‐to‐high SSIM values across systems (Figure 6). However, systematic differences remain, particularly in image sharpness, noise characteristics, and intensity uniformity. Quantitative image comparison was not performed for EPI scans on Scanner 5 (Vendor B) and the phantom EPI scan on Scanner 7 (Vendor C) due to differing matrix sizes. Vendor‐specific observations are summarized below.

**Vendor A**: High visual consistency is observed between Pulseq–Gadgetron and vendor‐native implementations (Figures 4 and 5), reflected by relatively low NRMSE values (mostly ∼20%) and relatively high SSIM (∼70%–95%) (Figure 6). For MPRAGE, Scanners 1–3 show slight intensity differences in phantom scans; for in vivo scans on Scanner 1, vendor‐native images exhibit higher white‐matter intensity than Pulseq–Gadgetron counterparts. For EPI, Scanner 4 shows the largest deviations, including mild differences in intensity, edge sharpness, and contrast, consistent with elevated NRMSE/HFEN and reduced SSIM. Pulseq–Gadgetron echo‐planar images yield lower SNR than the vendor‐native counterparts.

**Vendor B**: For MPRAGE, phantom scans show good agreement between Pulseq–Gadgetron and vendor‐native images (NRMSE 21%, HFEN 40%, and SSIM 88%). In contrast, in vivo scans exhibit larger discrepancies. Vendor‐native images show reduced apparent brain tissue intensity, reflected in worse quantitative metrics (NRMSE 73%, HFEN 113%, and SSIM 50%). This is due to the absence of fat suppression in the vendor's sequence (Table 3). The resulting high scalp fat signal alters automatic intensity scaling, reducing dynamic range for brain tissue. As shown in Figure S2, thresholding to remove fat signal rescales brain intensity and improves apparent agreement with Pulseq–Gadgetron images, confirming that differences stem from fat handling rather than brain tissue encoding. For EPI, vendor‐reconstructed images (phantom SNR = 244; in vivo SNR = 195) appear smoother and less noisy than the Pulseq–Gadgetron images (phantom SNR = 209; in vivo SNR = 171), consistent with k‐space zero‐padding (from 80 × 80 to 128 × 128) and Fermi filtering applied in the vendor's reconstruction pipeline but absent in Gadgetron.

**Vendor C**: For MPRAGE, vendor‐native images on Scanner 6 exhibit different noise characteristics, intensity uniformity, and gray–white matter contrast from Pulseq–Gadgetron images, reflected in a low phantom SSIM (45%) and a high in vivo HFEN (85%). These differences likely arise from vendor‐optimized SENSE reconstruction, which leverages accurate coil sensitivity maps and integrated B0/B1 field corrections, whereas the GRAPPA‐based Gadgetron pipeline does not. The hyperintense signal observed within cerebral arteries at 7 T on Scanner 7 (Figure 4) is attributed to a combination of factors: the prolonged T1 relaxation time of blood at ultra‐high field and the inflow of incompletely or non‐inverted arterial blood when using a local transmit head coil. These effects collectively alter the vascular contrast observed with the MPRAGE sequence. Echo‐planar brain images at 7 T also exhibit greater geometric distortion than at 3 T, consistent with stronger susceptibility‐induced B0 inhomogeneities at higher field strengths (Figure 5). Additionally, vendor‐reconstructed echo‐planar phantom images are smoother due to interpolation (from 80 × 80 to 192 × 192).

**Vendor D**: For Scanners 8 and 9, phantom MPRAGE images show good agreement on visual inspection, as supported by the high SSIM (74% and 86%, respectively), low NRMSE (24% and 25%), and moderate HFEN (61% and 64%). Vendor‐native images appear to have slightly better resolution and contrast than Pulseq–Gadgetron counterparts. In vivo, vendor‐native MPRAGE images appear slightly noisier, consistent with higher NRMSE (33% and 42%, respectively) and HFEN (76% and 75%, respectively), and lower SSIM (63% and 65%, respectively). These differences arise from variations in noise decorrelation and k‐space filtering: these operations were disabled in the vendor‐native reconstruction environment but were performed in the Gadgetron pipeline.

Overall, our Pulseq–Gadgetron workflow produces images that are visually comparable to vendor‐native results across scanners and vendors (Figures 3, 4, 5), with moderate‐to‐high SSIM typically ranging from 60% to 95% (Figure 6). Residual differences are primarily observed in high‐frequency image features, including edge sharpness, noise texture, and intensity uniformity, as reflected by relatively high HFEN (∼40%–120%) and, in some cases, elevated NRMSE (∼30%–50%).

These observations underscore that, even with detailed natural‐language protocol descriptions and meticulous console configurations, vendor‐native implementations can introduce subtle but measurable variability in both sequence execution and image reconstruction across platforms. In contrast, in the proposed workflow, Pulseq enables highly consistent sequence execution while Gadgetron ensures standardized reconstruction based on the MRD data standard across platforms, thereby enhancing reproducibility in multi‐site, cross‐vendor MRI studies.

## 4 Discussion

Reproducibility in MRI remains a persistent challenge due to variability in data acquisition, formatting, and reconstruction across sites and vendors. To address this, we developed an open‐source workflow that harmonizes all three stages by extending the Pulseq framework with advanced features. This workflow was successfully validated using MPRAGE and EPI protocols across nine scanners spanning two magnetic field strengths, five independent sites, and four major vendors.

For data acquisition, we provided comprehensive documentation specifying all key sequence parameters (e.g., repetition time, echo time, field of view, and readout bandwidth), as well as MATLAB scripts for configuring vendor‐native and Pulseq‐based sequences, respectively. Despite extensive efforts by MR experts at each site to match the provided parameters in vendor‐based configurations, substantial system‐specific variability persisted, for example, in readout bandwidths and fat‐suppression implementations (Table 3). This underscores that natural‐language instructions and manual console configuration are insufficient for achieving harmonized sequence execution across platforms. In contrast, the Pulseq‐based configuration required only minor, fully transparent modifications to the MATLAB source code to accommodate scanner‐specific constraints and spatial‐encoding conventions (Table 2) for sequence execution via the corresponding Pulseq interpreters. Therefore, Pulseq preserved consistency in nearly all implementation details (e.g., RF pulse shapes, gradient strengths, and slew rates) across platforms, demonstrating its potential to enhance reproducibility of data acquisition in cross‐vendor, multi‐site MRI studies.

For data formatting and sharing, all participating sites provided Pulseq‐generated raw k‐space data in MATLAB format (.*mat*) in acquisition order, along with the corresponding Pulseq sequence file (.*seq*) rather than vendor‐specific proprietary formats. These datasets were standardized into the vendor‐independent MRD format (.*h5*), leveraging the LABELs and Sequence Definitions in the .*seq* file to embed the necessary reconstruction metadata. This approach eliminates reliance on proprietary data structures, streamlines data sharing and reuse, and fosters transparent communication across sites and vendors.

For vendor‐integrated online reconstruction, all sites were instructed to minimize console‐based post‐processing (e.g., distortion correction and image filtering) to reduce vendor‐specific variability across platforms. Despite these efforts, differences in reconstruction algorithms persisted. For example, Vendor B applied a Fermi‐shaped smoothing filter and EPI k‐space zero‐padding, while Vendor C employed image‐space‐based parallel imaging (SENSE) for MPRAGE reconstruction, in contrast to the k‐space‐based parallel imaging approaches (GRAPPA/ARC) implemented by other vendors (Table 3). Tracking and harmonizing such differences remain challenging because vendor‐based reconstruction pipelines operate as proprietary “black boxes” with no accessible source code for their internal steps (e.g., coil sensitivity estimation, B0/B1 calibration, intensity bias correction, etc.). These findings demonstrate that manual protocol adjustments within vendor‐integrated environments are insufficient to achieve harmonized image reconstruction across platforms. In contrast, Gadgetron reconstructs standardized MRD data using transparent, publicly accessible pipelines, thereby eliminating vendor‐specific barriers. Its capability for harmonized and reproducible image reconstruction has also been rigorously demonstrated in our prior work on an automated, open‐source, vendor‐independent quality assurance protocol [39].

Several practical challenges remain in integrating Pulseq‐based sequences with vendor‐integrated reconstruction pipelines for real‐time image display on the scanner console. First, implementing vendor‐integrated online reconstruction algorithms for a given pulse sequence typically requires extensive low‐level programming within proprietary development environments, demanding substantial time and effort. Second, these environments are usually closed‐source, fixed software solutions that may not readily support novel pulse sequences (e.g., those using non‐Cartesian k‐space trajectories) and often lack the flexibility to support extensible, customizable post‐processing. Third, harmonizing vendor‐integrated reconstruction algorithms across software versions and vendors is inherently difficult due to potentially divergent solutions across platforms and closed‐source pipelines.

Gadgetron can help mitigate these challenges by providing transparent, customizable, and harmonized reconstruction via its shared libraries and toolboxes for Pulseq‐based MRD data. However, in this work, all Gadgetron reconstructions were performed offline, requiring manual raw data export and MRD conversion, which slows the innovation cycle in both research and clinical translation. For online reconstruction, we enabled Pulseq–ICE integration on Vendor A's platforms using the extended Pulseq framework, which properly sets measurement data headers via LABELs and Sequence Definitions for vendor‐based reconstruction. Additionally, Gadgetron online reconstruction was achieved on Vendor A's Scanner 2 (VE11C) in Site 1 through ICE integration [49, 52]. Nonetheless, Gadgetron installation for online use remains technically demanding—especially across software versions—and is currently limited to Vendor A. An alternative approach is Pulseq integration with OpenRecon, which streams Pulseq‐based data to a Docker container for reconstruction and post‐processing using custom research algorithms or third‐party tools (e.g., Gadgetron, BART [53]), and then returns processed images to the host for real‐time display. While OpenRecon installation is relatively straightforward, it is manufacturer‐provided and therefore restricted to Vendor A and specific software versions (XA51 or higher). Together, these limitations underscore the urgent need for an easy‐to‐install, vendor‐independent, open‐source, and customizable framework for online reconstruction. Such a tool would accelerate innovation, facilitate multi‐site harmonization, and bridge the gap between research and clinical translation.

In this work, we learned and demonstrated how to achieve data acquisition harmonization across scanners using Pulseq, summarizing key considerations in Table 2. Through this multi‐site, multi‐vendor MRI initiative, we identified several practical factors critical for achieving consistent data acquisition across platforms. First, scanner models and vendors may vary in their spatial‐encoding conventions due to variations in the relationships between gradient‐induced phase offsets and encoded spatial locations, as well as differences in coordinate system handedness. For example, for EPI, Vendor B required an RF phase offset opposite to that of Vendor A to achieve feet–head slice selection, whereas Vendor D required inversion of the phase‐encoding gradient polarity relative to Vendor A to achieve anterior–posterior encoding. Prior knowledge of these conventions is therefore essential, and a standardized test pipeline should be developed to systematically characterize them across platforms. Second, scanners differ in hardware and timing constraints, such as automatic readout oversampling and RF dead time. Consequently, a Pulseq‐based sequence that executes successfully on one scanner may fail on another. Unlike typical multi‐site studies that achieve harmonization through multiple iterative adjustments across vendor‐native platforms, our project started with a fixed Pulseq reference sequence that was then adapted to each vendor's specific constraints. While this approach transparently revealed scanner‐specific hard limits that required minor modifications (Table 2), it also meant that harmonization could not be achieved solely through iteration of high‐level parameters. To address this challenge and reduce the need for scanner‐specific code modifications in future studies, we recommend a community‐driven effort to collect and share hardware specifications across scanners, enabling sequence design within common operational limits from the outset. Third, identical sequence execution may result in physical and/or physiological differences across different systems. For example, at 7 T, cerebral vasculature can exhibit fundamentally different contrast behavior than at 3 T. Hyperintense arterial signal is better understood as a field‐dependent vascular contrast mechanism, arising from prolonged blood T1 relaxation at ultra‐high field in combination with inflow of incompletely or non‐inverted arterial blood when using a local transmit head coil (Figure 4) [54]. This should be distinguished from susceptibility‐induced signal loss and geometric distortion, which are also amplified at 7 T but are most prominent near tissue–air interfaces, such as the inferior frontal and temporal lobes (Figure 5). These distinct field‐dependent effects may require targeted adjustments to sequence parameters and interpretation to ensure consistent performance across field strengths. In practice, Pulseq‐based sequences should be tailored to the scanner hardware, study populations, research objectives, and other factors, to ensure robust, reproducible performance.

Developing open‐source, modular, and portable pipelines to minimize sources of variability has become an important focus in MRI research [55]. Veldmann et al. introduced an open‐source end‐to‐end workflow integrating Pulseq for data acquisition with BART [53] for reconstruction, validated on Vendors A and B [56]. In this framework, Pulseq‐acquired raw data can be streamed to a reconstruction server either offline or online; however, online streaming requires Vendor A‐specific FIRE [57] integration, and the workflow depends on a computationally intensive metadata generation step for MRD conversion. Karakuzu et al. proposed another vendor‐neutral workflow that utilizes the proprietary RTHawk [58] platform for fully transparent sequence development, reconstruction, and post‐processing [4]. Raw k‐space data were stored and annotated in MRD format, and the exported images were organized according to the Brain Imaging Data Structure (BIDS) [59] standard. This pipeline improved inter‐vendor reproducibility of quantitative MRI across scanners from Vendors A and B. In contrast, our workflow eliminates the need for separate metadata annotation by leveraging LABELs and Sequence Definitions. Moreover, it has been validated on four major vendors (Vendors A–D), and relies exclusively on fully open‐source, vendor‐independent, non‐proprietary tools—Pulseq, Gadgetron, and MRD—that are freely accessible and user‐friendly, thereby further lowering barriers to adoption.

However, several practical factors should be considered when adopting this workflow. Basic familiarity with MATLAB or Python is the primary expertise required. The open‐source Pulseq toolbox (MATLAB version [34]) and PyPulseq [40] (Python version) are released under the permissive MIT license and are freely accessible online, supported by tutorials [60] and additional materials on the Pulseq GitHub website that guide users through sequence development from scratch. Regarding technical specifications, Gadgetron can be installed via Conda or Docker without dedicated server infrastructure, while the MRD format can be read and built in MATLAB or Python with no additional software [61, 62]. As for licensing bottlenecks, the vendor‐specific Pulseq interpreter is obtainable from its developers, subject to vendor approval (e.g., the Siemens interpreter under a C2P agreement). Step‐by‐step instructions, including software installation and guidance on obtaining the interpreter, are available online [51].

A limitation of the present study is the inability to directly compare inter‐vendor reproducibility between vendor‐native and Pulseq‐based protocols using identical phantoms or traveling subjects due to geographic constraints. The primary goal was to demonstrate the feasibility—consistent acquisition, formatting, and reconstruction across nine scanners from four vendors—which our results confirm as achievable, with vendor‐native images showing visual comparability and reliability. However, without a standardized phantom or traveling‐subject cohort, we could not quantitatively assess the extent to which our workflow improves harmonization for data pooling or estimate the statistical power gains for multi‐site reproducibility. Specifically, it remains unclear whether observed inter‐vendor differences exceed typical inter‐subject variability (a key benchmark for biological relevance), and phantom‐only metrics may underestimate true biological variability (e.g., physiological noise, motion, and positioning). Addressing these gaps—quantifying the reduction in inter‐site variance achieved by our workflow versus vendor‐native approaches—remains an important goal for future traveling‐subject studies.

There are also limitations in comparing Pulseq–Gadgetron images with vendor‐native images. First, quantitative assessment of EPI‐specific artifacts, such as ghosting or geometric distortion, was precluded by the lack of standardized phantoms across sites. Second, the observed differences on Vendor C reflect not only acquisition harmonization but also differences in parallel imaging models (GRAPPA vs. SENSE). Disentangling these contributions would require implementing SENSE within Gadgetron with equivalent coil sensitivity maps, which was not feasible due to unavailable calibration data and the lack of Cartesian SENSE support in Gadgetron. Even with pre‐scan data, replicating Vendor C's SENSE reconstruction is challenging, as its proprietary implementation differs substantially from standard SENSE [63] described in the literature or implemented in open‐source tools.

We advocate for an open‐source, fully accessible, and inclusive research ecosystem for the entire MR community. This approach spans the full research pipeline, including hardware design [31, 64], data acquisition [14, 65, 66], simulation [67, 68, 69], data management [6, 59], image reconstruction [35, 53, 56, 57, 70, 71, 72], data processing and analysis [73], research dissemination, and beyond [7, 74, 75]. This ecosystem strengthens reproducibility, collaboration, and accessibility for students and early‐career researchers through educational initiatives [76, 77, 78]. Furthermore, it has enabled on‐site construction of an open‐source, low‐cost, low‐field MR system in a low‐income country [79], demonstrating its potential to improve MRI accessibility and sustainability worldwide. Open‐source practices also benefit individual researchers through increased visibility, citations, collaborations, and funding opportunities [80, 81]. Given these advantages, broader adoption might be expected, underscoring the need for continued advocacy.

## 5 Conclusions

We established an open‐source workflow that standardizes data acquisition, formatting, and reconstruction using the advanced Pulseq framework, and successfully validated this workflow across nine scanners, encompassing two magnetic field strengths, five independent sites, and four major vendors. Our work demonstrates the workflow's feasibility for achieving transparent, reproducible MRI across sites and vendors. More broadly, this work underscores the value of an open, accessible, and inclusive research ecosystem—one that not only fosters education and global collaboration within the MR community but also strengthens professional opportunities for individual researchers.

## Funding

This work was supported by research grants NIH R01 EB032378, NIH U24 NS120056, and DFG INST 39 1365‐1. This research was funded by the European Union under Horizon Europe Grant Agreement 1011078393, MRITwins. This research is also a part of project 22HLT02 A4IM, that has received funding from the European Partnership on Metrology, co‐financed from the European Union's Horizon Europe Research and Innovation Programme and by the Participating States.

## Conflicts of Interest

Yuhai Xie and Jiayu Zhu are employed by Shanghai United Imaging Healthcare Co. Ltd., Shanghai, China. Zheng Liu is employed by Wuhan United Imaging Life Science Instrument Co. Ltd., Wuhan, China. Qingping Chen and Maxim Zaitsev maintain a research collaboration with Siemens Healthineers (Erlangen, Germany). The other authors declare no conflicts of interest.

## Supporting information


**Figure S1:** Quantitative analysis of image differences for MPRAGE and EPI in phantom and in vivo datasets across all four of Vendor A's scanners (Scanners 1–4). Each panel shows evaluation metrics grouped by scanner model. Colors denote comparison types (A–E), while circular and square markers indicate phantom and in vivo measurements, respectively. SSIM values were calculated within the phantom or head. HFEN = high‐frequency error norm; NRMSE = normalized root mean squared error; SSIM = structural similarity index.
**Figure S2:** Effect of thresholding on vendor‐native MPRAGE images from Vendor B. The “Original” vendor‐native (“Prod+Prod”) images shows high subcutaneous fat signal due to the absence of fat‐suppression module in the vendor's MPRAGE sequence. After thresholding (“Thresholded” row), subcutaneous fat signal is reduced and the remaining brain tissue signal is automatically rescaled, increasing gray/white matter intensity. This rescaling improves the apparent agreement between vendor‐native and Pulseq–Gadgetron images.
**Table S1:** Quantitative evaluation of phantom and in vivo MPRAGE images across all platforms. **A (B)**: Type‐A (Type‐B) comparison between Pulseq‐based and vendor‐integrated acquisitions for **ICE‐based (Gadgetron‐based)** images from Vendor A; **C (D)**: Type‐C (Type‐D) comparison between Gadgetron and ICE reconstructions for **product‐sequence‐based (Pulseq‐based)** images from Vendor A; **E**: Type‐E comparison between vendor‐native and Pulseq‐based, Gadgetron‐reconstructed images. HFEN = high‐frequency error norm; NRMSE = normalized root mean squared error; Phan. = Phantom; SSIM = structural similarity index metric.
**Table S2:** Quantitative evaluation of phantom and in vivo echo‐planar images across all platforms. **A (B)**: Type‐A (Type‐B) comparison between Pulseq‐based and vendor‐integrated acquisitions for **ICE‐based (Gadgetron‐based)** images from Vendor A; **C (D)**: Type‐C (Type‐D) comparison between Gadgetron and ICE reconstructions for **product‐sequence‐based (Pulseq‐based)** images from Vendor A; **E**: Type‐E comparison between vendor‐native and Pulseq‐based, Gadgetron‐reconstructed images. HFEN = high‐frequency error norm; NRMSE = normalized root mean squared error; Phan. = Phantom; SSIM = structural similarity index metric.

## Data Availability

The data that support the findings of this study are openly available in Zenodo at https://doi.org/10.5281/zenodo.20725134.
