# Supplementary material for: An Open‐Source, Reproducible MRI Data Acquisition and Reconstruction Workflow Using Pulseq: Multi‐Site and Cross‐Vendor Validation
Source: Magn Reson Med. 2026 Aug 6;96(6):2721–38. doi: 10.1002/mrm.70548 (PMC13619859; doi:10.1002/mrm.70548)
Supplement: Supplementary file 1 [file MRM-96-2721-s001.docx]

**Figure S1.** Quantitative analysis of image differences for MPRAGE and EPI in phantom and *in vivo* datasets across all four of Vendor A’s scanners (Scanners 1–4). Each panel shows evaluation metrics grouped by scanner model. Colors denote comparison types (A–E), while circular and square markers indicate phantom and *in vivo* measurements, respectively. SSIM values were calculated within the phantom or head. Abbreviations: NRMSE = normalized root mean squared error; SSIM = structural similarity index; HFEN = high-frequency error norm.

**Figure S2.** Effect of thresholding on vendor-native MPRAGE images from Vendor B. The “Original” vendor-native (“Prod+Prod”) images shows high subcutaneous fat signal due to the absence of fat-suppression module in the vendor’s MPRAGE sequence. After thresholding (“Thresholded” row), subcutaneous fat signal is reduced and the remaining brain tissue signal is automatically rescaled, increasing gray/white matter intensity. This rescaling improves the apparent agreement between vendor-native and Pulseq–Gadgetron images.

**Table S1.** Quantitative evaluation of phantom and *in vivo* MPRAGE images across all platforms. **A** **(B)**: Type-A (Type-B) comparison between Pulseq-based and vendor-integrated acquisitions for **ICE-based (Gadgetron-based)** images from Vendor A; **C (D)**: Type-C (Type-D) comparison between Gadgetron and ICE reconstructions for **product-sequence-based (Pulseq-based)** images from Vendor A; **E**: Type-E comparison between vendor-native and Pulseq-based, Gadgetron-reconstructed images. Phan. = Phantom. NRMSE = normalized root mean squared error; SSIM = structural similarity index metric; HFEN = high-frequency error norm.

| **Scanner** | **Comparison** | **NRMSE (%)** | | **SSIM (%)** | | **HFEN (%)** | |
| --- | --- | --- | --- | --- | --- | --- | --- |
|  |  | **Phan.** | ***In vivo*** | **Phan.** | ***In vivo*** | **Phan.** | ***In vivo*** |
| **#1** | **A** | 12.0 | 12.5 | 89.8 | 81.9 | 27.7 | 30.0 |
|  | **B** | 10.0 | 11.7 | 92.4 | 87.5 | 25.8 | 26.9 |
|  | **C** | 27.0 | 28.3 | 77.2 | 70.0 | 57.6 | 68.2 |
|  | **D** | 24.5 | 28.0 | 77.2 | 70.1 | 55.4 | 67.5 |
|  | **E** | 19.6 | 28.3 | 88.6 | 71.2 | 33.1 | 59.7 |
| **#2** | **A** | 11.5 | 26.7 | 92.4 | 58.8 | 27.6 | 75.7 |
|  | **B** | 13.6 | 28.8 | 92.8 | 60.1 | 26.3 | 71.3 |
|  | **C** | 21.3 | 22.5 | 80.9 | 76.2 | 52.8 | 60.6 |
|  | **D** | 21.6 | 22.0 | 80.4 | 75.7 | 51.0 | 58.6 |
|  | **E** | 14.0 | 21.9 | 92.1 | 70.7 | 27.5 | 54.2 |
| **#3** | **A** | 11.6 | 18.0 | 92.5 | 79.7 | 26.6 | 51.0 |
|  | **B** | 17.5 | 18.7 | 91.3 | 84.0 | 28.3 | 36.2 |
|  | **C** | 19.2 | 21.0 | 81.1 | 78.4 | 50.9 | 61.7 |
|  | **D** | 21.8 | 21.7 | 80.0 | 76.8 | 48.6 | 58.0 |
|  | **E** | 16.7 | 22.1 | 90.5 | 78.6 | 28.7 | 57.4 |
| **#4** | **A** | 17.3 | 20.8 | 94.9 | 92.6 | 33.8 | 35.4 |
|  | **B** | 14.8 | 16.7 | 95.5 | 94.5 | 27.7 | 32.1 |
|  | **C** | 31.1 | 28.1 | 85.8 | 85.7 | 56.7 | 69.8 |
|  | **D** | 28.8 | 29.1 | 86.4 | 86.8 | 55.6 | 67.6 |
|  | **E** | 20.3 | 20.5 | 94.2 | 91.5 | 33.4 | 44.5 |
| **#5** | **E** | 21.0 | 73.2 | 87.9 | 49.9 | 40.3 | 113.4 |
| **#6** | **E** | 24.6 | 42.6 | 44.5 | 71.0 | 42.6 | 84.8 |
| **#7** | **E** | 56.4 | 48.7 | 72.3 | 57.5 | 104.3 | 73.7 |
| **#8** | **E** | 24.4 | 33.4 | 74.1 | 62.8 | 60.8 | 76.1 |
| **#9** | **E** | 25.3 | 42.1 | 85.9 | 64.1 | 63.6 | 74.5 |

**Table S2.** Quantitative evaluation of phantom and *in vivo* echo-planar images across all platforms. **A** **(B)**: Type-A (Type-B) comparison between Pulseq-based and vendor-integrated acquisitions for **ICE-based (Gadgetron-based)** images from Vendor A; **C (D)**: Type-C (Type-D) comparison between Gadgetron and ICE reconstructions for **product-sequence-based (Pulseq-based)** images from Vendor A; **E**: Type-E comparison between vendor-native and Pulseq-based, Gadgetron-reconstructed images. Phan. = Phantom. NRMSE = normalized root mean squared error; SSIM = structural similarity index metric; HFEN = high-frequency error norm.

| **Scanner** | **Comparison** | **NRMSE (%)** | | **SSIM (%)** | | **HFEN (%)** | |
| --- | --- | --- | --- | --- | --- | --- | --- |
|  |  | **Phan.** | ***In vivo*** | **Phan.** | ***In vivo*** | **Phan.** | ***In vivo*** |
| **#1** | **A** | 18.0 | 14.2 | 92.1 | 87.8 | 27.7 | 33.0 |
|  | **B** | 37.7 | 13.8 | 71.0 | 88.8 | 58.3 | 31.3 |
|  | **C** | 37.0 | 21.8 | 68.5 | 90.7 | 58.5 | 24.9 |
|  | **D** | 15.4 | 22.3 | 93.8 | 90.0 | 23.3 | 26.5 |
|  | **E** | 25.2 | 21.4 | 84.7 | 81.1 | 38.8 | 38.9 |
| **#2** | **A** | 21.1 | 9.6 | 90.4 | 95.6 | 31.8 | 21.9 |
|  | **B** | 16.3 | 8.7 | 92.9 | 96.1 | 29.0 | 19.6 |
|  | **C** | 18.6 | 20.4 | 91.5 | 88.7 | 34.0 | 33.2 |
|  | **D** | 25.7 | 23.5 | 88.6 | 86.0 | 42.4 | 39.1 |
|  | **E** | 21.7 | 22.4 | 87.9 | 87.5 | 36.9 | 36.8 |
| **#3** | **A** | 22.8 | 8.9 | 90.1 | 96.4 | 31.3 | 19.3 |
|  | **B** | 21.7 | 14.5 | 88.4 | 91.3 | 34.5 | 33.4 |
|  | **C** | 11.9 | 14.7 | 96.2 | 93.1 | 20.9 | 23.0 |
|  | **D** | 27.0 | 22.0 | 89.4 | 85.4 | 42.2 | 42.0 |
|  | **E** | 23.8 | 23.5 | 86.2 | 84.0 | 39.6 | 46.7 |
| **#4** | **A** | 11.9 | 11.4 | 95.5 | 96.2 | 18.8 | 16.9 |
|  | **B** | 12.1 | 13.5 | 93.7 | 93.0 | 18.8 | 24.1 |
|  | **C** | 53.3 | 44.0 | 67.6 | 84.6 | 69.4 | 43.2 |
|  | **D** | 55.6 | 45.3 | 61.9 | 75.0 | 81.4 | 56.3 |
|  | **E** | 51.8 | 51.5 | 64.8 | 75.5 | 77.0 | 56.3 |
| **#5** | **E** | - | - | - | - | - | - |
| **#6** | **E** | 47.6 | 28.0 | 60.8 | 76.3 | 68.5 | 46.6 |
| **#7** | **E** | - | 62.5 | - | 21.7 | - | 156.0 |
| **#8** | **E** | 39.1 | 32.2 | 76.2 | 54.2 | 62.1 | 78.8 |
| **#9** | **E** | 19.6 | 13.9 | 76.5 | 90.8 | 45.9 | 29.5 |
